# The impact of over-distraction on adjacent segment pathology and cage subsidence in anterior cervical discectomy and fusion

**DOI:** 10.1038/s41598-023-44998-4

**Published:** 2023-10-28

**Authors:** Lan-Li Hsueh, Yu-Cheng Yeh, Meng-Ling Lu, Chi-An Luo, Ping-Yeh Chiu, Po-Liang Lai, Chi-Chien Niu

**Affiliations:** 1 Department of Orthopedic Surgery, Chang Gung Memorial Hospital, Linkou Branch, No.5, Fuxing St., Guishan Dist., Taoyuan City 333, Taiwan, ROC; 2 Bone and Joint Research Center, Chang Gung Memorial Hospital, Linkou Branch, No.5, Fuxing St., Guishan Dist., Taoyuan City 333, Taiwan, ROC; 3 College of Medicine, Chang Gung University, No. 259, Wenhua 1st Rd., Guishan Dist., Taoyuan City 333, Taiwan, ROC; 4 Department of Orthopedic Surgery, Chang Gung Memorial Hospital, Kaohsiung Branch, No. 123, DAPI Rd., Niaosong Dist, Kaohsiung City 833, Taiwan, ROC

**Keywords:** Diseases, Medical research, Risk factors, Signs and symptoms

## Abstract

Over-distraction has been shown to be a risk factor for cage subsidence and postoperative neck pain after anterior cervical discectomy and fusion (ACDF). Biomechanical studies have demonstrated increased adjacent segment intradiscal pressure after ACDF. The purpose of this study is to determine if over-distraction of the index disc has an effect on adjacent segment pathology. A consecutive series of 145 patients who received primary ACDF for cervical degenerative pathologies from January 2010 to December 2017 were retrospectively reviewed. The patients were divided into: (1) Over-distraction group (postoperative–preoperative index disc height ≥ 2 mm), and (2) No-distraction group (postoperative–preoperative index disc height < 2 mm). Outcome measures included radiographic parameters, Japanese Orthopaedic Association (JOA) score, and incidences of cage subsidence, radiological and clinical adjacent segment pathologies (RASP and CASP) were compared between the two groups preoperatively, postoperatively, and at the final follow-up. The two groups were comparable with respect to age, follow-up length, JOA score, incidence of CASP, and radiographic parameters. The Over-distraction group (83 patients; 115 levels) had smaller preoperative index disc height (4.5 vs. 5.2 mm, p < 0.001), but taller postoperative index disc height (7.7 vs. 6.6 mm, p < 0.001) than No-distraction group (62 patients; 90 levels) Furthermore, significantly higher incidences of cage subsidence (47% vs. 31%, p = 0.04) and RASP (any progression: 48% vs. 15%, p < 0.001; progress ≥ 2 grades: 25% vs. 7%, p = 0.001) were observed in the Over-distraction group. The multivariate analysis indicated that over-distraction and multilevel fusion were independent risk factors for RASP. There were no clinical outcome differences between the Over-distraction group and the No-distraction group in ACDF. Over-distraction of the index level of ≥ 2 mm should be avoided because it significantly increases the incidences of RASP and cage subsidence.

## Introduction

Anterior cervical discectomy and fusion (ACDF) has been proven to be a safe and effective treatment for cervical degenerative pathologies since its first introduction by Smith and Robinson^1^. With consistent clinical results and surgical prognosis, the procedure includes complete removal of the disc materials and protruding osteophytes, and replacement with autologous bone grafts, allografts, interbody spacers, or a combination of these. Various materials have been applied as interbody spacer. Polyetheretherketone (PEEK) cages have been widely applied due to good biocompatibility and radiolucency allowing fusion assessment^1,2^. On the other hand, porous tantalum trabecular metal (TM) cages are famous of high porosity and biomechanical similarity of elastic modulus to bone^3,4^. Other materials of interbody spacer were also applied but not in our study. The elastic modulus of carbon fiber composite frame cages are close to bone, which helps to decrease the stress shielding and to promote bony fusion as described in Wolff's law^5^. Titanium cage subsidence with the collapse of disc height and kyphotic deformity had been observed in previous study^6^.

Adjacent segment pathology (ASP) remains to be one of the major concerns after ACDF, with a reported annual incidence of 2.9%^7^. ASP can be further divided into clinical adjacent segment pathology (CASP) and radiological adjacent segment pathology (RASP), based on the presentation of correlated clinical symptoms or not^8^. Common manifestations of CASP include radiculopathy, myelopathy and axial neck pain. Several surgical strategies have been proposed to lower the incidence of ASP, such as the motion-preserved total disc arthroplasty, minimizing damage to the adjacent intervertebral discs and anterior longitudinal ligament, and restoring normal cervical sagittal alignment^9^.

Cage subsidence is another complication that potentially has negative effects on the surgical outcomes^10,11^. Decreased foraminal height, local kyphosis, and compromise of cervical sagittal alignment thus occur after the cage sinks into the vertebral body^12,13^. Although anterior plating has been advocated to increase the stability and the fusion rate, postoperative dysphagia, ASP due to injury of the anterior longitudinal ligament, and lack of micro-motion in the bone-graft interface due to rigid fixation have all been reported with anterior plate augmentation of ACDF^14,15^.

To restore the index disc height, spinal surgeons tend to select larger sized grafts/cages during ACDF. However, large cage size (> 5.5 mm) has been proposed as a risk factor for cage subsidence^16^. Furthermore, Yamagata et al.^11^ reported that the patients with cage subsidence had a significantly greater distraction ratio than those without cage subsidence. Our research aims to investigate how Over-distraction affects the radiographic parameters, cage subsidence, adjacent segment pathology and clinical outcomes in patients undergoing ACDF surgery.

## Material and methods

### Patients

This retrospective study analyzed a consecutive series of patients presented with myelopathy or radiculopathy due to cervical degenerative pathologies and received primary anterior cervical discectomy and fusion (ACDF) between January 2010 and December 2017 at our institute.

The inclusion criteria were: (1) Received 1 to 2 levels primary ACDF; 2) Received follow-up for at least 24 months. The patients were excluded if any of the following criteria were met: 1) Underwent surgery due to non-degenerative pathologies including traumatic injury, malignant tumor and infection; (2) Received ACDF for more than 3 levels; (3) Received cervical disc prosthesis; (4) Revisional cervical spine surgeries; (5) Received anterior plate augmentation, and (6) Follow-up < 24 months.

This study was conducted at Chang Gung Memorial Hospital, Taiwan. This study was approved with informed consent waiver by the Chang Gung Medical Foundation Institutional Review Board (IRB No. 201900795B0). All methods were performed in accordance with the relevant guidelines and regulations.

### Surgical techniques

After introduction of general anesthesia and endotracheal intubation, patients were placed in the supine position with the neck in mild extension. Target vertebral bodies were exposed via a standard Smith-Robinson anterior cervical approach. The operative levels were checked by intraoperative portable X-ray. A Casper distractor was applied for distraction of the vertebral bodies. All disc materials, including the herniated fragments, posterior osteophytes, and ossification of posterior longitudinal ligament (OPLL) were then removed under the assistance of a surgical microscope. The vertebral endplates were prepared with meticulous curettage to avoid endplate damages. Adequate neural decompression was confirmed through partial removal of the posterior longitudinal ligament.

The cages were packed with artificial bone substitutes, such as tricalcium phosphate, and/or demineralized bone matrix (DBM), and/or morselized allograft. After trials of size selection, the cages were inserted into the disc space. All patients wore a cervical collar for 6 weeks postoperatively.

### Assessment of results

All patients received clinical and radiographic evaluations before surgery, immediate after surgery, and 6 weeks, 3, 12, 24 months after surgery until the last follow-up. Successful fusion was defined as the presence of bridging trabecular bone, or a < 2 mm distance change of the index level spinous process tips in plain radiographs dynamic series. Radiological adjacent segment pathology (RASP) was assessed on plain radiograph findings according to the grading proposed by Hilibrand et al.^7^, and modified by Chung et al.^17^. Table 1 presents the grading system for RASP, which is divided into six grades. All patients with symptoms related to cervical spine, including neck pain, arm pain, cervical myelopathy symptoms or other radiculopathy symptoms were arranged MRI at outpatients department to check if there are progressive adjacent segment pathologies or not. Patients with symptoms correlated to MRI findings of adjacent segment pathologies were diagnosed with clinical adjacent segment pathology (CASP). Cage subsidence was defined as a loss of index level disc height > 3 mm as compared to the height measured on the immediate postoperative radiographs^10^. The Japanese Orthopedic Association (JOA) scores for cervical spine were evaluated before and after surgery, and at the final follow-up. Radiographic parameters measured on plain radiographs before and after surgery, and at the final follow-up included index level disc height, and the heights of the cranial adjacent and caudal adjacent levels, local Cobb angle at the index level. Other measurements included cervical sagittal parameters including C2-C7 cervical lordosis (CL), T1 slope (TS), T1 slope minus cervical lordosis (TS–CL), neck tilt, thoracic inlet angle, and cervical sagittal vertical axis (cSVA). Two spine fellows evaluated each radiograph separately and made their own interpretations. The averaged measurements were set as final results for continuous data like disc height or Cobb angle. For categorical data, similar opinions between the two spine fellows were directly recorded as final results. The senior surgeon was consulted for final decision if the opinions were different between the two spine fellows.

**Table 1:** Radiological adjacent segment pathology (RASP) grading on plain radiographs.

Grade*	Plain radiograph findings
0	Normal
I	Hypertrophy of the uncinate process
IIA	Ossification to < 50% of disc height
IIB	Ossification to < 50% of disc height + disc space narrowing
IIIA	Ossification to ≥ 50% of disc height
IIIB	Ossification to ≥ 50% of disc height + disc space narrowing
IV	Complete bridging of the adjacent disc space
V	Disc space narrowing to > 50% of normal disc height + posterior osteophyte or displacement

*The grading system was proposed by Hilibrand et al.^7^ and modified by Chung et al.^17^.

### Statistical analysis

Previous study showed interbody height increases 2.2 mm in high torque group and 1.9 mm in low torque group^18^. Recent biomechanical study had reported that larger-sized interbody cages (the height of ≥ 2 mm of the index disc height) could result in remarkable variations in biomechanical responses of adjacent levels^19^. To evaluate the effect of over-distraction, the patients were divided into two groups: (1) Over-distraction group (postoperative index disc height–preoperative index disc height ≥ 2 mm), and (2) No-distraction group (postoperative index disc height–preoperative index disc height < 2 mm). Patient demographic data and outcome measures were analyzed and compared between the two groups. Statistical differences between the two groups were calculated using the independent T test and the chi-square test. Risk factors of progression of RASP were determined by univariate and multivariate binary logistic regression analysis. A *p* value < 0.05 was considered statistically significant. Receiver operator characteristic (ROC) curves and the corresponding area under the curve (AUC) were used to evaluate how the prediction model of RASP preformed. All statistical analyses were performed using SPSS version 25 software (IBM-SPSS, Armonk, NY).

## Results

### Data demographics

**Figure 1:**
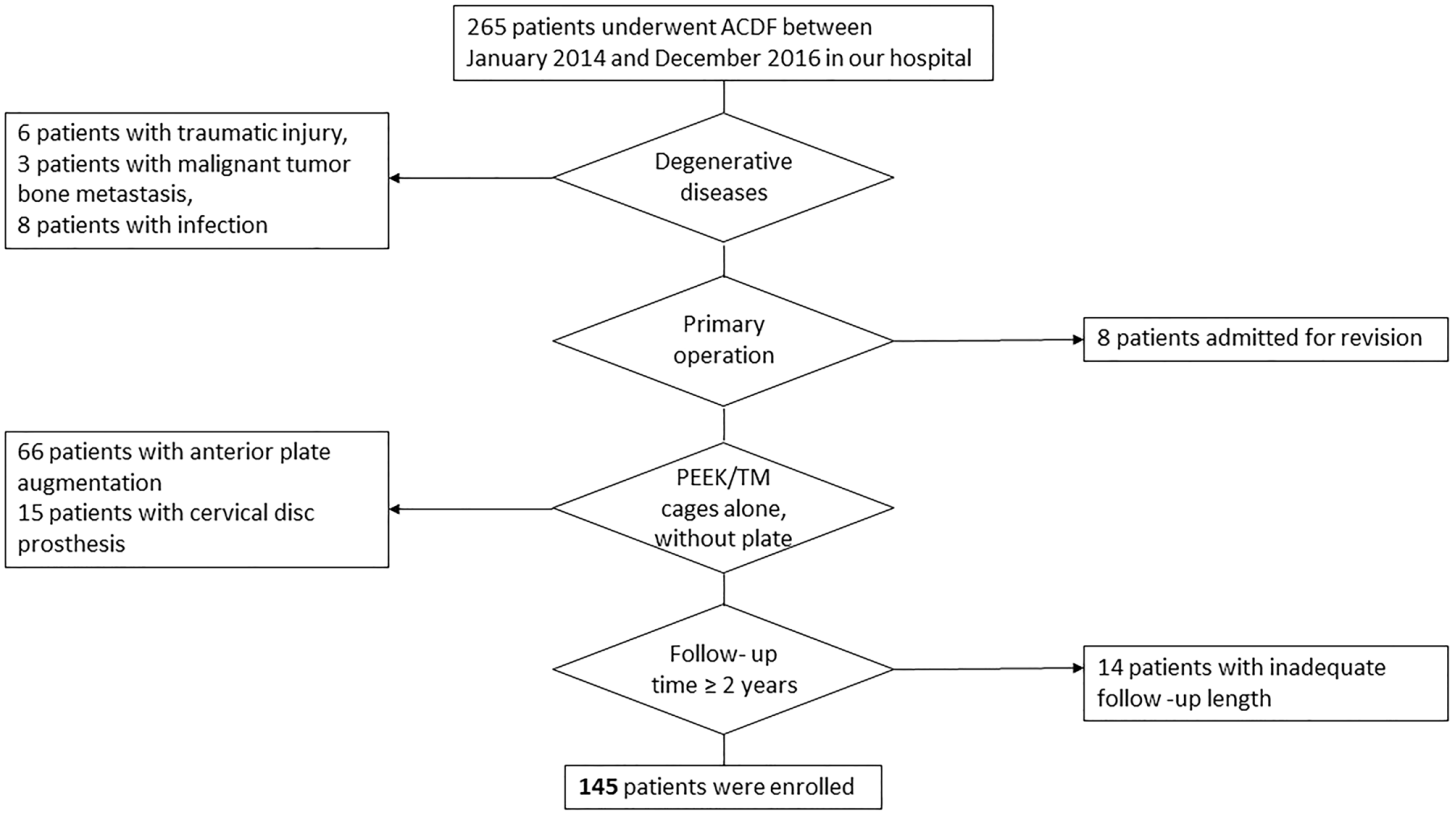
Flowchart of patient identification.

A total of 265 patients underwent ACDF between 2014 and 2016 were reviewed and the review process was showed in Fig. 1. There were 17 patients underwent the surgery for non-degenerative diseases and 8 patients were revision cases. Sixty-six patients were excluded for anterior plating and fifteen patients chose cervical disc prosthesis. For minimum 2 years follow-up, 14 patients were excluded. Finally, a total of 145 patients (88 males, 57 females) implanting with 67 levels of TM cages and 139 levels of PEEK cages were included in this study. For all patients, the mean age at the time of surgery was 53.7 years (range 31–78 years), and the mean follow-up length was 32 months (range 24–105 months). (Table 2) Preoperative diagnoses included herniation of intervertebral disc (HIVD), spondylosis with osteophyte formation, and OPLL.

**Table 2:** Patient demographic and clinical data.

Number of patients	145
Age (years)	53.7 ± 11.9
Sex (M/F)	88/57
Total fused levels	206
Follow-up length (months)(SD)	32 ± 17
Neurological symptoms	
Myelopathy	53/145 (37%)
Radiculopathy	92/145 (63%)
Fused levels	
1	84/145 (58%)
2	61/145 (32%)
Cage materials	
TM	48/145 (67%)
PEEK	97/145 (33%)
Successful fusion	128/145 (88%)
RASP	49/145 (34%)
CASP	7/145 (5%)
Cage subsidence	58/145 (40%)
JOA score (SD)	
Preoperative	11.5 ± 2.1
Postoperative	13.8 ± 2.0
Final follow-up	14.4 ± 2.0

Age, follow-up length, and JOA scores were presented as mean ± standard deviation.

M: male; F: female; RASP: radiological adjacent segment pathology; CASP: clinical adjacent segment pathology; JOA score: Japanese Orthopedic Association score.

Successful fusion was achieved in 88% (182/206) of the implanted levels. The overall incidence of cage subsidence was 36% (75/206). RASP was observed in 34% (49/145) of the patient; however, only 7 of the patients developed symptoms making the incidence of CASP = 5% (7/145).

### Comparisons of outcome between the two groups

There were 83 patients included in the Over-distraction group and 62 in the No-distraction group. The age, number of fused levels, follow-up length, and fusion rate were comparable between the two groups (Table 2). The incidence of RASP was significantly higher in the Over-distraction group (48% vs. 15%, *p* < 0.001). In addition, the incidence of moderate progression of RASP (≥ 2 grades) was greater in the Over-distraction group (21% vs. 4%, *p* = 0.001).

**Figure 2:**
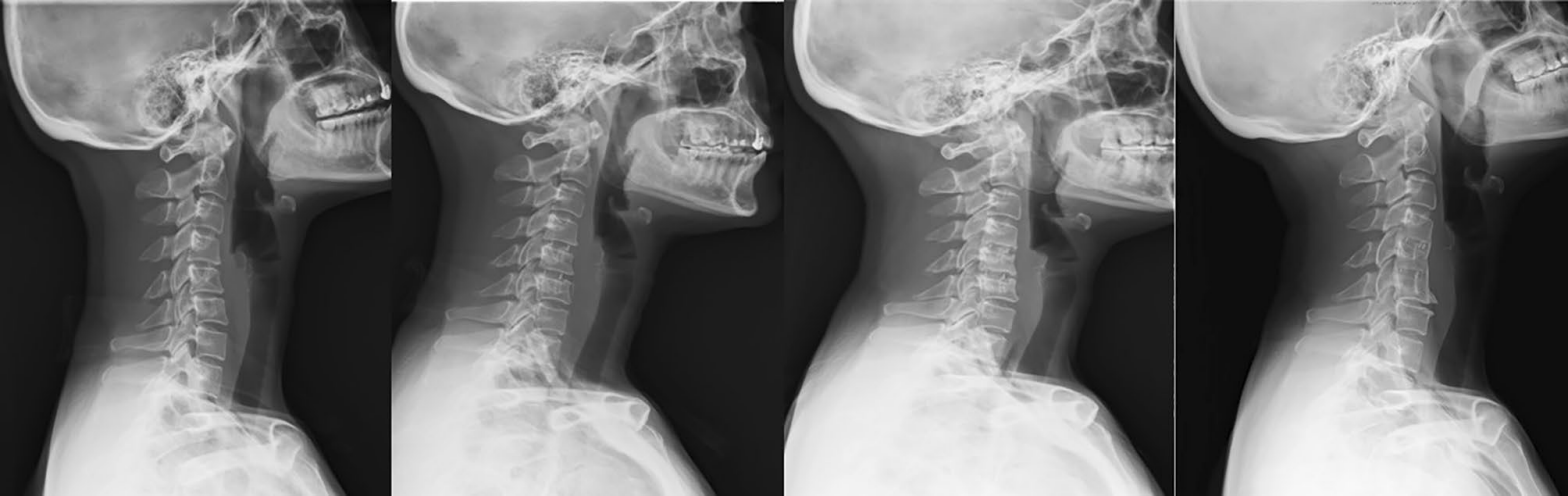
A 41-year-old female suffered from severe neck pain with bilateral hands numbness for more than one year. 2a. C-spine lateral view done before the operation, which showed C4-5-6 HIVD and spondylosis. 2b. Postoperative C-spine lateral view showed C4-5-6 ACDF with PEEK cage was done. 2c. Well fusion without obvious RASP at 1 year after the operation. Clinical symptoms improved significantly. 2d. Patient complained posterior neck pain with left arm radiation pain and numbness over C7 dermatome for 3 months. C-spine lateral view showed adjacent segment disc degeneration. This patient was classified into the CASP group.

All seven cases of CASP were in the Over-distraction group and the incidence was significantly higher than the No-distraction group (8% vs. 0%, p = 0.02). Figure 2 showed one case who underwent C4-5-6 ACDF with PEEK cage suffered from CASP 5 years after the operation. There were three patients received revision surgery after primary ACDF within follow up. Two of the patients suffered from pseudoarthrosis and one of the patients suffered from severe clinical adjacent segmental pathology.

The incidence of cage subsidence (disc height loss > 3 mm) was significantly higher in the Over-distraction group than the No-distraction group (47% vs. 31%, *p* = 0.04). The JOA scores were comparable between the two groups preoperatively, postoperatively, and at the final follow-up.

### Comparisons of radiographic parameters between the two groups

Radiographic parameters of the two groups at different time points are summarized in Table 3, and illustrated in Fig. 2. All these data was measured by the two spine fellows. The interrater agreement of the parameter measurements was excellent with an ICC of 0.907, (95% CI 0.822–0.950). The Over-distraction group had a significantly smaller preoperative index disc height (4.5 vs. 5.2 mm, *p* < 0.001), but higher postoperative index disc height (7.7 vs. 6.6 mm, *p* < 0.001) and local Cobb angle (9.8° vs. 7.7°, *p* = 0.04) than the No-distraction group. However, at the final follow-up, the index disc height (4.8 vs. 4.5 mm, *p* = 0.35) and local Cobb angle (5.2° vs. 5.0°, *p* = 0.86) was similar between the two groups. All the other radiographic parameters except postoperative cSVA (25.8 vs. 19.8 mm, *p* = 0.03) were comparable between the two groups preoperatively, postoperatively, and at the final follow-up, respectively and was showed at Table 4.

**Table 3:** Comparison of outcomes between the Over-distraction and No-distraction groups.

	Over-distraction	No-distraction	*p* value
Patient number	83	62	
Age (years old)	53.7 ± 10.6	53.6 ± 13.6	0.94
Sex (M/F)	40/22	48/35	0.49
Total fused levels	116	90	
Fused levels			0.61†
1	50 (60%)	34 (63%)	
2	33 (40%)	28 (31%)	
Cage materials			0.02†
TM	34 (41%)	14 (23%)	
PEEK	49 (59%)	48 (77%)	
Follow-up length (months)	33 ± 17	30 ± 16	0.34*
Successful fusion	74/83 (89%)	54/62 (87%)	0.79†
RASP			
Any progression	40/83 (48%)	9/62 (15%)	< 0.001†
Moderate ($\ge$ 2 grades)	21/83 (25%)	4/62 (7%)	0.001†
CASP†	7/83 (8%)	0/62 (0%)	0.02†
Cage subsidence	39/83 (47%)	19/62 (31%)	0.04†
JOA score (SD)			
Preoperative	11.4 ± 2.1	11.8 ± 2.2	0.26*
Postoperative	13.8 ± 2.1	13.7 ± 2.0	0.88*
Final follow-up	14.5 ± 2.1	14.4 ± 1.9	0.83*

*Mann–Whitney U-test.

^†^Chi-square test.

Age, follow-up length, and JOA score were presented as mean ± standard deviation.

M: male; F: female; RASP: radiological adjacent segment pathology; CASP: clinical adjacent segment pathology; JOA score: Japanese Orthopedic Association score.

**Table 4:** Comparison of radiographic parameters comparisons between the Over-distraction and No-distraction groups.

Radiographic parameters	Over-distraction	No-distraction	*p* value*
Index disc height (mm)			
Preoperative	4.5 ± 1.1	5.2 ± 1.0	< 0.001
Postoperative	7.7 ± 1.1	6.6 ± 0.9	< 0.001
Final follow-up	4.8 ± 1.9	4.5 ± 1.8	0.35
Cranial adjacent disc height (mm)			
Preoperative	5.7 ± 1.0	5.9 ± 0.9	0.38
Postoperative	6.0 ± 1.0	6.0 ± 0.8	0.90
Final follow-up	5.8 ± 1.0	6.0 ± 0.8	0.28
Caudal adjacent disc height (mm)			
Preoperative	5.5 ± 1.3	5.6 ± 1.2	0.53
Postoperative	5.8 ± 1.4	5.5 ± 1.2	0.23
Final follow-up	5.4 ± 1.3	5.5 ± 1.1	0.76
Local Cobb angle			
Preoperative	3.5° ± 5.8°	5.2° ± 5.2°	0.07
Postoperative	9.8° ± 6.4°	7.7° ± 5.0°	0.04
Final follow-up	5.2° ± 5.3°	5.0° ± 4.7°	0.86
C2–C7 cervical lordosis			
Preoperative	13.6° ± 10.0°	13.0° ± 10.2°	0.75
Postoperative	15.2° ± 10.0°	14.0° ± 8.9°	0.47
Final follow-up	14.6° ± 8.8°	13.1° ± 8.7°	0.30
T1 slope			
Preoperative	25.2° ± 7.2°	24.1° ± 8.6°	0.41
Postoperative	28.0° ± 8.3°	25.3° ± 8.1°	0.05
Final follow-up	25.0° ± 9.2°	24.0° ± 9.6°	0.54
TS–CL			
Preoperative	11.6° ± 10.3°	11.0° ± 9.8°	0.74
Postoperative	12.9° ± 9.8°	11.3° ± 8.3°	0.29
Final follow-up	10.4° ± 10.1°	11.0° ± 9.6°	0.74
Neck tilt			
Preoperative	26.8° ± 31.9°	34.5° ± 31.1°	0.14
Postoperative	48.9° ± 10.9°	50.1° ± 15.4°	0.59
Final follow-up	49.9° ± 11.5°	51.9° ± 11.0°	0.28
Thoracic inlet angle			
Preoperative	76.6° ± 10.7°	74.0° ± 16.3°	0.28
Postoperative	77.0° ± 11.6°	75.5° ± 17.8°	0.54
Final follow-up	74.9° ± 11.7°	75.9° ± 13.1°	0.62
cSVA (mm)			
Preoperative	19.7 ± 14.7	22.3 ± 16.8	0.34
Postoperative	25.8 ± 19.6	19.8 ± 11.2	0.03
Final follow-up	17.3 ± 12.4	18.9 ± 20.3	0.56

*Mann–Whitney U-test.

Data are presented as mean ± standard deviation.

TS: T1 slope; CL: C2-C7 cervical lordosis; cSVA: cervical sagittal vertical axis.

### Risk factors of RASP

Results of the univariate and multivariate logistic regression analysis to determine risk factors for RASP are shown in Table 5. Significant risk factors in the univariate analysis were over-distraction, multilevel fusion, TM cage implantation, smaller preoperative neck tilt, longer follow-up length and smaller preoperative local cobb angle. Multivariate analysis revealed over-distraction (Odds ratio [OR] = 7.52; 95% confidence interval [CI]: 2.82–20.04) and multilevel fusion (OR = 2.52; 95% CI 1.12–5.68) were the independent risk factors for RASP.

**Table 5:** Univariate and multivariate analysis for risk factors of RASP.

	OR	95% CI	*p* value
Univariate analysis			
Over-distraction	5.58	2.24–12.76	< 0.001
Multilevel fusion	2.50	1.52–4.12	< 0.001
TM cage	3.55	1.74–7.25	0.001
Pre-OP neck tilt	0.99	0.98–1.00	0.012
Follow-up length	1.03	1.01–1.05	0.017
Pre-OP local Cobb angle	0.93	0.87–0.99	0.022
Age	0.99	0.96–1.02	0.538
Multivariate analysis			
Over-distraction	7.52	2.82–20.04	< 0.001
Multilevel fusion	2.52	1.12–5.68	0.025
TM cage	0.93	0.07–12.48	0.957
Pre-OP neck tilt	1.01	0.97–1.04	0.701
Follow-up length	1.02	1.00–1.05	0.123

RASP: radiological adjacent segment pathology; CI: confidence interval; OR: odds ratio.

**Figure 3:**
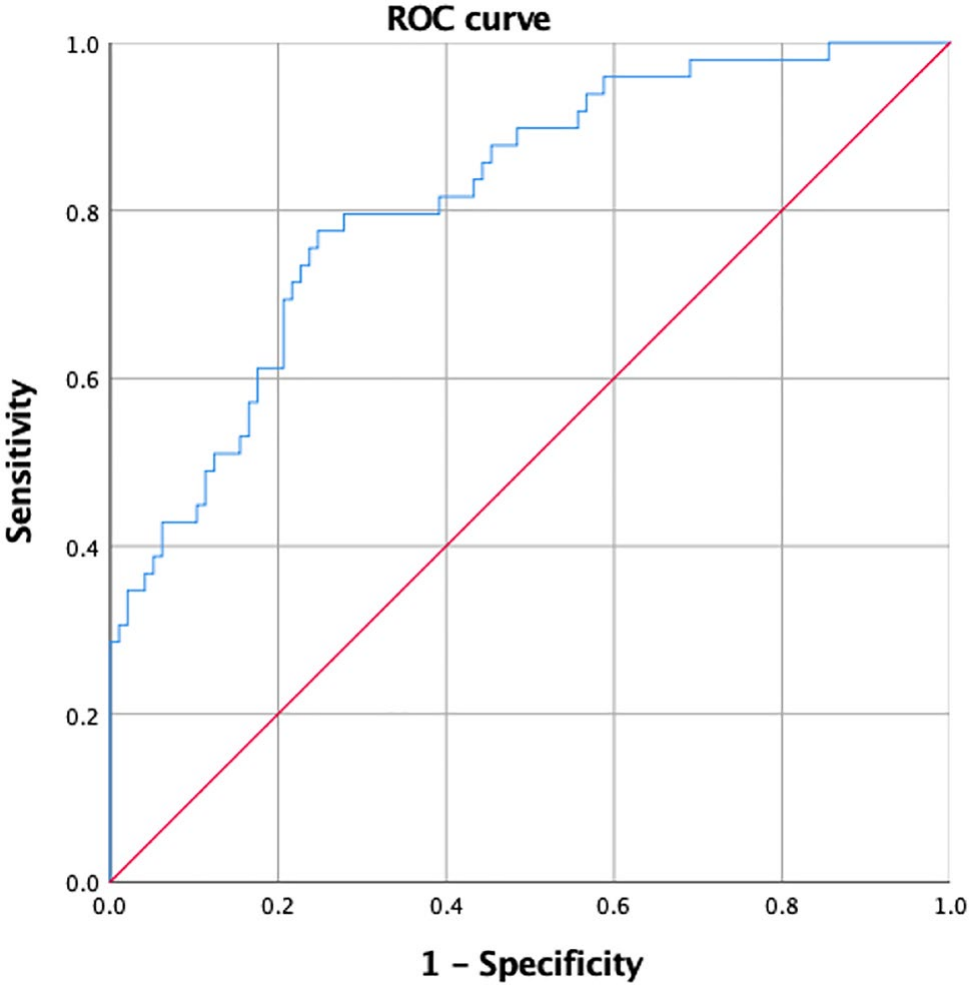
Receiver operator characteristic (ROC) curve analysis of the model for predicting radiological adjacent segment pathology (RASP) developed from multivariate logistic regression analysis. The area under the ROC curve AUC = 0.815 (95% CI 0.744–0.887).

The ROC curve of the prediction model of RASP developed from the multivariate logistic regression analysis is shown in Fig. 3, and the AUC = 0.815 (95% CI 0.744–0.887). This prediction model of RASP development has good efficacy. Over-distraction and multilevel fusion can cause higher risk of RASP progression in ACDF.

## Discussions

Porous tantalum cages and polyetheretherketone(PEEK) cages were widely applied for ACDF. Porous tantalum cages have been reported similar biomechanical properties to that of cancellous bone, which allows good load transfer and minimizes the stress-shielding effect^3^. PEEK is chemically inert and does not allow for protein absorption and promotion of cell adhesion and bone contact when compared to titanium. Both TM cages and PEEK cage resulted in high fusion rate in cervical spine and lumbar spine. However, higher cage subsidence rate in TM cages than PEEK cages was revealed^2^. Therefore, we included patients who received ACDF with TM cages or PEEK cages in this study, and the overall fusion rate (88%) was consistent with prior reports^20–22^.

Distraction of the intervertebral disc space allows better visualization when removing the disc materials and posterior osteophytes. However, over-distraction with excessive force can result in injuries to the facet joints at the index level^23^. Kirzner et al.^24^ reported that over-distraction of the facet joint of ≥ 3 mm is associated with worse functional outcomes and pain scores in patients with traumatic cervical injuries treated with ACDF. Furthermore, an excessive distraction force might increase the pressure of the adjacent discs and the stress of the adjacent structures. Yuan et al.^25^ investigated the influences of the height of disc arthroplasty on cervical biomechanics using finite element study. Protheses of ≥ 2 mm of the index disc height significantly increased the adjacent intradiscal pressure, adjacent facet joint forces, and bone-implant interface stresses of the protheses, as compared to prostheses of < 2 mm of the index disc height. Interestingly, with or without excessive distraction, our results showed that the index disc heights in both groups had collapsed to a similar degree at the final follow-up (Fig. 2B). The physiologic tension of surrounding musculo-ligamentous structures might play a role in determining the final index disc height in ACDF with grafts/cages. On the other hand, Aryan et al.^26^ proposed relaxing the distraction force after discectomy because they showed that the same degree of distraction could be achieved with 20N less of initial forces after removal of intervertebral discs. Since excessive distraction force might interfere the physiologic tension and affect the selection of graft/cage size, we suggested releasing the distraction force during graft/cage size trials to avoid selecting over-size grafts/cages based on the results of this study.

Risk factors for the development of ASP identified in prior studies include young age, high T1 slope, pre-existing disc degeneration, disruption of adjacent soft tissue, plate placement close to the adjacent disc, and poor sagittal profile^9,15,27,28^. Several surgical strategies have been proposed to prevent ASP, including motion-preserved disc arthroplasty^29^, minimizing damages to adjacent level structures^15,28^, and restoring normal cervical sagittal alignment^30^. Hilibrand et al.^7^ demonstrated that multilevel ACDF had a significantly lower risk of CASP than single-level ACDF. On the other hand, Basques et al.^31^ reported no significant differences in the development of RASP between different fusion lengths of ACDF. However, You et al.^32^ reported that patients with ASP after ACDF had longer fusion length than patients without ASP. Recent study showed that using larger-sized interbody cages can result in remarkable variations in biomechanical responses of adjacent levels, which may indicate as risk factor for adjacent segment disease^19^. In our study, the Over-distraction group had a significantly higher incidence of RASP progression than the No-distraction group (any progression: 48% vs. 15%, *p* < 0.001; progress ≥ 2 grades: 25% vs. 7%,* p* = 0.001). Over-distraction (OR = 7.52; 95% CI 2.82–20.04) and multilevel ACDF (OR = 2.52; 95% CI 1.12–5.68) were identified as independent risk factors for RASP progression after ACDF surgery. (Table 5) All seven patient with CASP progression were found in the Over-distraction group. The incidence of CASP was significantly higher in the Over-distraction group than in the No-distraction group (8% vs. 0%, *p* = 0.02).

Cage subsidence after ACDF is one of the major concerns for spinal surgeons. One systemic review reported that the mean incidence of cage subsidence after ACDF was 21%, and ranged from 0 to 83%^10^. Cage subsidence can lead to pseudoarthrosis, segmental instability, progressive kyphotic deformity, and loss of foraminal height^33,34^. However, cage subsidence might not necessarily lead to poor clinical outcomes^13^, which may be due to segmental kyphosis with preserved posterior disc height, and maintenance of global cervical alignment^12^. The relation between larger sized cages and cage subsidence was first described by Yamagata et al.^11^ Cage subsidence occurred in 12 (19%) out of 63 levels (47 patients) in patient receiving fusion with stand-alone titanium cages, and the distraction ratio was significantly higher in patients with cage subsidence than those without. They also reported that a cage size of 6.5/7.5 was associated with a significantly higher risk of cage subsidence than a cage size of 4.5/5.5 (50% vs. 15%, *p* = 0.037). Igarashi et al.^16^ demonstrated a significant correlation between cage height and subsidence (*p* < 0.01) in a series of 78 patients (105 levels) received stand-alone ACDF with a minimum follow-up of 1 year. They found that titanium cages were associated with a greater degree of subsidence than PEEK cages (2.26 mm vs. 1.27 mm, *p* < 0.01), and there was no difference in the amount of subsidence between titanium cages and PEEK cages when the cage height was < 5 mm. However, contradictory results regarding the incidence of cage subsidence, irrespective of cage size or height, have also been reported^34,35^. Recent studies have indicated that subsidence and fusion rate were comparable between TM cage and PEEK cage^36^. However, different ratio of the implant’s surface area to the bone surface area may also affect the cage subsidence rate^37^. In our study, the Over-distraction group had a significant higher incidence of cage subsidence (disc height loss > 3 mm) than the No-distraction group (47% vs. 31%, *p* = 0.04).

The major limitations of this study include the retrospective nature, and the relatively small patient cohort size. The radiographic follow-up was only based on plain radiographs, but not based on computer tomography (CT) or magnetic resonance imaging (MRI). The general indications for anterior plate augmentations of ACDF in our department were for patients with cervical spondylolisthesis or received ACDF for more than 3 levels. Furthermore, the radiographic parameters of the overall cervical alignment, e.g. C2-C7 lordosis and cSVA, were easily affected by the head postures. In this study, the patients were asked to maintain horizontal gaze with their personal ease when taking radiographs. In addition, JOA score focused more on cervical myelopathy symptoms than radiculopathy symptoms. Different functional score such as NDI score, can measure self-rated disability due to neck pain. VAS score is widely used to quantify pain. Various functional score should be applied to help us understand the exact clinical outcome. Further prospective studies with a long-term follow-up were needed to make definite conclusions regarding the effect of over-distraction to ACDF.

## Conclusions

In conclusion, Over-distraction of the index level of ≥ 2 mm significantly increases the incidence of RASP, CASP and cage subsidence. Based on these findings, we suggest releasing the distraction force during the graft/cage size selection in ACDF to avoid the possibility of over-distraction of disc height, which may lead to a higher incidence rate of RASP, CASP and cage subsidence.

## Data Availability

The data used in this study cannot be made publicly available due to privacy concerns. The data contain sensitive or identifiable information that must be protected in accordance with institutional policies and applicable laws and regulations. However, data are available from the authors upon reasonable request and with the appropriate legal and ethical approvals. Interested researchers may contact niuchien@cgmh.org.tw to request access to the data.
